# Interference of oleamide with analytical and bioassay results

**DOI:** 10.1038/s41598-020-59093-1

**Published:** 2020-02-07

**Authors:** Urška Jug, Katerina Naumoska, Valentina Metličar, Anne Schink, Damjan Makuc, Irena Vovk, Janez Plavec, Kurt Lucas

**Affiliations:** 1 Department of Food Chemistry, National Institute of Chemistry, Hajdrihova 19, 1001 Ljubljana, Slovenia; 2 Faculty of Chemistry and Chemical Technology, University of Ljubljana, Večna pot 113, 1000 Ljubljana, Slovenia; 3 Multiphase Chemistry Department, Max Planck Institute for Chemistry, Hahn-Meitner-Weg 1, 55128 Mainz, Germany; 4 Slovenian NMR Centre, National Institute of Chemistry, Hajdrihova 19, 1001 Ljubljana, Slovenia; 5 EN-FIST Centre of Excellence, Trg Osvobodilne fronte 13, 1000 Ljubljana, Slovenia

**Keywords:** Immunology, Plant sciences, Chemistry

## Abstract

During sample preparation and analysis, samples are coming in contact with different labware materials. By four unrelated analytical (phytochemical and pharmaceutical) case-studies and employing different analytical techniques, we demonstrated the potential misinterpretation of analytical results due to the use of contaminants-leaching labware during sample handling. Oleamide, a common polymer lubricant and a bioactive compound, was identified as a main analytical interference, leaching from different labware items into solvents, recognised as chemically compatible with the tested polymer material. Moreover, anti-inflammatory effect of oleamide at 100 μg mL^−1^ and considerable pro-inflammatory effect of the plastic syringe extractables (containing oleamide) at the same level were shown in a TLR4-based bioassay. Taking these results into account, together with the fact that oleamide can be a compound of natural origin, we would like to notify the professional public regarding the possible erroneous oleamide-related analytical and bioassay results due to the use of oleamide-leaching labware. Researchers are alerted to double check the real source of oleamide (labware or natural extract), which will prevent further reporting of false results. Analysis of procedural blanks with *de-novo* developed UHPLC-ESI-MS method is, among some other strategies, proposed for detection of oleamide interference and avoidance of misleading results of certain analyses.

## Introduction

Due to the convenient use, plastic equipment is omnipresent in analytical and biological laboratories. Plastic additives, which enhance polymer properties, prolong their shelf-life and increase their functionality, are inevitably used during its production^1^. Among the known lubricants and slip additives, fatty acid amides are recognised, including oleamide, erucamide and stearamide^2,3^. Due to the lower number of carbon atoms, oleamide migrates faster from the polymer compared to erucamide and stearamide^2^.

Moreover, oleamide is a bioactive signaling molecule found in cerebrospinal fluid of sleep-deprived animals^4,5^. It is reported to affect cannabinergic CB-1^6^, GABA_A_ and serotonergic 5-HT receptors^7,8^ and to possess anti-inflammatory activity^9–13^. Various other pharmacological effects of oleamide such as cannabinoid like behaviour^6,14^, inhibition of the enzyme human monoamine oxidase B^8,15^, closure of gap-junctions^16^ and activation of TRPV1 vanilloid receptors^16^ are also communicated. Despite the already reported studies regarding bioassay interferences^8,15,17^ from plastic labware leachables, including oleamide, it has been continuously reported as analyte in natural extracts^5,11,12,18–21^. At the same time, it has been rarely double checked to be a possible plastic labware interference^22–25^, perhaps due to its natural origin. For instance, few fatty acid amides (myristamide, palmitamide, linoleamide, elaidamide, stearamide and erucamide), frequently used as lubricating agents in polymer industry^26^, were reported to be the responsible compounds for the toxicity of the alga *Prymnesium parvum*^19^. These results may or may not represent a coincidence. However, the possibility for interferences of plastic-leaching contaminants was not considered. Moreover, due to oleamide’s bioactivity, the reliability of the published bioassay guided fractionation studies, leading to discovery of oleamide^11,12^ has never been questioned. Therefore, we feel the urge to notify the professional public on the continuous reporting of possibly false positive results regarding oleamide as analyte. Moreover, we would like to alert analytical chemists about the need to double check the real origin of oleamide, thus preventing misinterpretation of analytical results and at the same time notify labware manufacturers regarding this issue. This will prevent future publication of erroneous data and aid to the quality and reliability of the future studies.

To demonstrate the analytical dilemma regarding oleamide as analyte or interference, four different and unrelated analytical (phytochemical and pharmaceutical) case studies using different techniques (HPTLC, HPTLC-ESI-MS, HPTLC-APCI-MS, DI-ESI-MS, UHPLC-ESI-MS and ^1^H NMR) are presented. Even though the four case-studies were independently performed (in the course of different projects, by different researchers, and some of them even in different laboratories), the conclusions drawn from the individual studies led to oleamide as a main analytical interference leaching from different labware. The next aim of the study was to develop a simple UHPLC-ESI-MS method, which will help researchers to identify oleamide-leaching labware. Due to oleamide’s proven activity in some cell-based bioassays, including anti-inflammatory effects^9–13^, experiments on TLR4 antagonistic activity aiming to show interferences in the biological response were performed as well.

In the light of this study, we would like to raise the question, whether the previously published results on a global scale, reporting identification of oleamide in samples (e.g. phytochemical extracts), are trustworthy. Moreover, we aim to tackle another problem concerning the reliability of the already published bioassay studies. Namely, oleamide can be present in labware itself, mostly made of plastic, and only careful analytical approach could eliminate this interference, as discussed in this paper.

## Materials and Methods

### Interference of oleamide with analytical results (Case study 1 – Case study 4)

#### Case-study 1: Multi-dimensional (HP)TLC fractionation of Japanese knotweed rhizome extract, followed by fraction structural elucidation by NMR

Japanese knotweed rhizome acetonic 70%_(aq)_ extract (acetone: Honeywell, North Carolina, USA) was fractionated on (HP)TLC plates (Merck, Darmstadt, Germany) in multiple dimensions to isolate (+)-catechin^27^. Fractions were extracted by methanol (LC-MS grade, Honeywell Reagents, Seelze, Germany) and ^1^H NMR spectrum (in CD_3_OD, Armar AG, Döttingen, Switzerland) was acquired for characterisation of the isolated compound. Extracts (procedural blanks) of different labware used during the isolation procedure, as well as of some alternative labware, were prepared and tested with ^1^H NMR and with an HPTLC method for separation of lipid classes^28^.

Extracts of different labware as follows: polyvinylidene fluoride (PVDF, 0.45 μm, d = 25 mm), PVDF (0.45 μm, d = 8 mm), hydrophilic polytetrafluoroethylene (H-PTFE, 0.20 μm, d = 8 mm) and regenerated cellulose (RC, 0.45 μm, d = 25 mm) membrane filters for syringe, 5 mL and 20 mL plastic syringes, membrane filter paper (PVDF type GV, 0.22 μm), 50 mL centrifuge vial and plastic Pasteur pipette were obtained after multiple extractions with methanol (LC-MS grade, Honeywell Reagents) (see Supplementary Information). Methanol as blank and amber glass storage vial with PTFE lined cap were also tested. During the extraction procedures, the use of other plastic labware was avoided.

For NMR analyses, the extracts obtained after multiple extractions of PVDF (d = 8 mm and d = 25 mm), H-PTFE (d = 8 mm) membrane filters attached to 20 mL plastic syringes, plastic Pasteur pipettes and plastic centrifuge vials (50 mL), all stored in amber glass storage vials with PTFE lined caps, were dried under N_2_. Methanol was also treated with N_2_ flow to concentrate possible impurities. Dry residues were dissolved in 0.5 mL of CD_3_OD or in case of 5 mL plastic syringe extract also in 0.5 mL CDCl_3_ (both obtained from Armar AG). CD_3_OD or CDCl_3_ were also used as lock solvents. Solvent residual peaks had a role of internal standards in case of CD_3_OD, while 0.03% tetramethylsilane (TMS), *v/v* was used as a reference standard for experiments performed in CDCl_3_. CD_3_OD blank in a glass NMR tube was also tested for potential impurities. ^1^H NMR spectra were acquired on Agilent Technologies DD2 600 MHz spectrometer equipped with a 5 mm HCN cold probe at 25 °C. The labware-leaching contaminant was identified on the basis of the characteristic NMR resonances which were assigned according to their chemical shifts (δ) expressed in ppm, multiplicity of signals, coupling constants (*J*) and their integrated intensity using MestReNova NMR software (Mestrelab, Santiago de Compostela, Spain).

**Figure 1:**
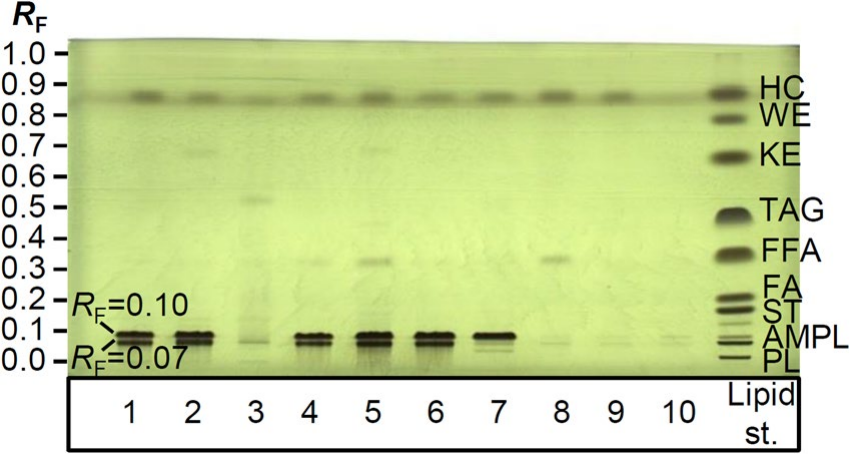
Chromatogram of extracts obtained after multiple extractions of labware: PVDF (d = 25 mm, **1**), PVDF (d = 8 mm, **2**), PVDF filter paper (**3**), H-PTFE (**4**), regenerated cellulose (**5**), plastic syringe (20 mL, **6**), plastic syringe (5 mL, **7**), plastic centrifuge vial (50 mL, **8**), plastic Pasteur pipette (**9**), methanol treated with N_2_ flow (**10**) and lipid standards mixture (**11**). HPTLC silica gel plate was developed according to the method for separation of lipid classes^28^ and documented under white light illumination after derivatisation with MoP. Legend: PL - phospholipids, AMPL - acetone-mobile polar lipids, ST - sterols, FA - fatty alcohols, FFA - free fatty acids, TAG - triacylglycerols, KE - ketones, WE - wax esters, HC - hydrocarbons.

For HPTLC analysis, all labware extracts obtained after multiple extractions and methanol blank, stored in amber glass storage vials with PTFE lined caps, were dried under N_2_ and the solid residues were dissolved in 0.5 mL methanol. Lipid standards mixture was prepared by dissolving the following lipids in chloroform (Merck), with mg mL^−1^ concentrations given in parenthesis: L-α-phosphatidylcholine (0.09), α-monopalmitin (0.25), cholesterol (0.12), cetyl alcohol (0.10), palmitic acid (0.22), tripalmitin (1.00), 3-hexadecanone (0.18), stearyl palmitate (0.11) and squalane (0.06). All lipid standards were of 99% purity and purchased from Sigma-Aldrich (Darmstadt, Germany), with exception of squalane, which was obtained from Merck^28^. Lipid standards mixture, labware methanolic extracts obtained after multiple extractions and methanol (treated with N_2_ flow) blank were applied on a 20 cm × 10 cm glass backed HPTLC silica gel 60 plate (Art. No. 1.05641.0001) by Linomat 5 (CAMAG, Muttenz, Switzerland) as 8 mm bands, 16 mm from the left edge and 5 mm from the bottom of the plate (Fig. 1). Applications on the plate were as follows: extracts of membrane filters for syringes, filter paper, syringes (all 25 μL), plastic centrifuge vial, plastic Pasteur pipette, methanol (treated with N_2_ flow) (all 64 μL) and lipid standard mixture (27 μL). The plate was developed using an HPTLC method for separation of different lipid classes^28^. For detection, the plate was immersed in 10% ethanolic (Carlo Erba, Val de Reuil Cedex, France) solution of molybdophosphoric acid hydrate (Merck) - MoP reagent^28,29^ for 2 seconds, dried in a stream of warm air and heated on TLC plate heater III (CAMAG) at 150 °C for 35 min. DigiStore 2 Documentation system (CAMAG) was used for capturing HPTLC plate images under white light illumination after post-chromatographic derivatisation.

HPTLC coupled to electrospray ionisation (ESI)-MS was used to analyse methanolic extracts of 5 mL and 20 mL plastic syringes (as prepared for HPTLC) using twice pre-developed (1^st^ methanol:formic acid (Merck) (10:1, *v/v*), 2^nd^ methanol)^30,31^ HPTLC silica gel plate (20 cm × 10 cm). Methanolic extracts (70 μL) were applied by Linomat 5 (CAMAG) as 50 mm band, 5 mm from the bottom of the plate. Methanolic standard solution of oleamide (50 μg mL^−1^, Sigma-Aldrich, 1.25 μg) was applied twice as 8 mm bands. Background MS signals were also analysed from the middle zone of the plate (Supplementary Fig. S1). The HPTLC method for separation of lipid classes^28^ was used for development of the plate. After plate development and drying, it was cut in three pieces and only side pieces were derivatised with MoP, as shown in Supplementary Fig. S1. TLC–MS interface (CAMAG) with oval elution head (4 mm × 2 mm) was used to elute the zones of interest from the plate into the MS. Heated ESI in positive mode ((+)HESI) was used to ionise the eluted compounds. MS parameters, mobile phase composition and flow rate are described in ‘UHPLC-ESI-MS’ section (below). A C18 (4 mm × 3 mm I.D., Phenomenex, Torrance, CA, USA) guard column and 0.5 μm in-line filter (Idex, Health & Science, Oak Harbor, WA, USA) were mounted before the MS (LTQ Velos MS, Thermo Fisher Scientific, Waltham, MA, USA) ion source. MS spectra were acquired in the *m/z* range of 50–2,000. For MS^n^ experiments, target precursor ions were fragmented using the collision energy of 35%. After HPTLC–MS^n^ analysis, the middle part of the plate was additionally derivatised with MoP to confirm the position accuracy of the eluted zones.

#### Case-study 2: HPLC fractionation of cinnamon extract and identification of the unknown analytes by direct injection (DI)-ESI-MS

Commercial 70%_(aq)_ ethanolic extract of cinnamon (*Cinnamomum verum*) was fractionated by HPLC^32^. The isolated fractions were tested in a bioassay and the potent ones were further analysed by HRMS and GC-MS to identify the compounds potentially responsible for the anti-inflammatory activity^32^. Prior to obtaining the final data^32^, problems with their DI-MS identification were encountered.

In the scope of troubleshooting, standard eugenol (0.5 μg mL^−1^ in ethanol, Fluka, Ph.Eur), certain active cinnamon fractions, blanks and extracts of labware, used during the experimental work (listed in Table 1, labelled ‘DI-MS’), were prepared by single extraction and separately tested with DI-MS. Different solvents (mobile phase, ethanol or acetonitrile) were tested as extraction media for labware and dissolution (solvation, dilution or concentration) media for the isolated cinnamon fractions with anti-inflammatory activity.

**Table 1:** Extracts of disposable laboratory materials obtained after single (**S**) or multiple (**M**) extractions and tested for presence of oleamide (*m/z* 282) with either DI-MS or LC-MS method. Extraction media and extraction procedure (S or M) were selected to obtain procedural blanks required in case-studies.

Method	Material tested	Extraction media	Oleamide	
			S	M
**Plastic laboratory materials**				
LC-MS	Pipette tip 1,000 µL **A**	MeOH	−	−
DI-MS	Pipette tip 200 µL **B**	m.p.	−	/
LC-MS	Centrifuge vial 15 mL **C**	MeOH	+	/
DI-MS	Centrifuge vial 15 mL **D**	m.p.	−	/
DI-MS	Centrifuge vial 15 mL **D**	ACN	−	/
LC-MS	Centrifuge vial 50 mL **C**	MeOH	−	+
LC-MS	Pasteur pipette **E**	MeOH	−	+
DI-MS	Pasteur pipette **F**	m.p.	+	/
LC-MS	Microcentrifuge tube 2 mL **G**	MeOH	−	−
LC-MS	Microcentrifuge tube with cellulose acetate filter 2 mL **H**	MeOH	−	/
DI-MS	Microcentrifuge tube with cellulose acetate filter 2 mL **H**	EtOH	−	/
LC-MS	PVDF syringe filter 0.45 µm **I**	MeOH	−	−
DI-MS	PVDF syringe filter 0.2 µm **J**	m.p.	−	/
LC-MS	H-PTFE syringe filter 0.2 µm **I**	MeOH	−	/
DI-MS	PTFE syringe filter 0.2 µm **K**	m.p.	−	/
LC-MS	Syringe 5 mL **L**	MeOH	+	+
LC-MS	Syringe 5 mL 26^th^ rinsing **L**	MeOH	+	/
LC-MS	Syringe 5 mL **L**	H_2_O	+	/
LC-MS	Syringe 20 mL **L**	MeOH	/	+
DI-MS	Syringe 5 mL **L**	m.p.	+	/
DI-MS	Syringe 5 mL **M**	m.p.	+	/
DI-MS	Syringe 5 mL **N**	m.p.	+	/
DI-MS	HPLC plastic vial insert **O**	ACN	−	/
DI-MS	HPLC plastic vial insert **O**	EtOH	−	/
**Other laboratory materials**				
LC-MS	HPLC glass vial insert with bottom-spring **J**	MeOH	+	/
LC-MS	HPLC glass vial insert **J**	MeOH	−	/
LC-MS	Glass syringe 5 mL **P**	MeOH	−	/
DI-MS	Glass syringe 1 mL **Q**	m.p.	−	/
LC-MS	Glass syringe 100 µL **Q**	MeOH	−	/
LC-MS	Glass Pasteur pipette **R**	MeOH	−	/
DI-MS	Glass Pasteur pipette **F**	m.p.	−	/
DI-MS	Glass fraction collector tube **O**	m.p.	−	/
LC-MS	MeOH (LC-MS grade) **S**	MeOH	−	−
LC-MS	Laboratory glove* **T**	MeOH	+	/

−/+ not detected/detected.

*Concentrated extract.

/not analysed.

m.p.mobile phase: 0.1% HCOOH_(aq)_:ACN (30:70, *v/v*).

A–T different manufacturers.

DI-MS analyses were performed on HPLC-MS system (Agilent 1200 Series-6130 Quadrupole) without column under isocratic elution using mobile phase composed of 0.1% formic acid_(aq)_ (Sigma-Aldrich) and acetonitrile (Fisher Scientific) in ratio 30:70, *v/v* and a flow rate of 0.2 mL min^−1^. ESI-MS method in positive mode, optimised with eugenol standard under flow injection analysis (FIA) was used for troubleshooting purposes. The MS parameters were as follows: drying gas flow and temperature 9 L min^−1^ and 340 °C, respectively, nebulizer pressure 10 psig, capillary voltage 5,000 V and fragmentor 85 V.

#### Case-study 3: HPTLC-APCI-MS analysis of Japanese knotweed leaves extract

Japanese knotweed leaves extract (see Supplementary Information) was analysed on a 20 cm × 10 cm glass backed C_18_ RP HPTLC plate (Merck). Separately, non-filtered and filtered blank (90% acetone_(aq)_:1 M triethylammonium acetate (TEAA) with pH 7^33^ in ratio 85:15, *v/v*, both obtained from Honeywell), standard oleamide (50 μg mL^−1^ in methanol, 1.25 μg) and filtered sample extract in duplicate were applied on another HPTLC plate of the same type. Solutions were applied on the plates as 8 mm bands, 15 mm from the left edge and 10 mm from the bottom edge by Linomat 5. The plates were pre-developed and developed using our method for separation of carotenoids^34^. Developed plates were dried under a stream of cool air, and only the second plate (except the last application) was derivatised by MoP (see case-study 1). Finally both plates were documented by DigiStore 2 documentation system under white light. TLC–MS interface, with oval elution head (4 mm × 2 mm) was used for elution of the zones of interest from the first HPTLC plate into MS system (LTQ Velos). A C18 (4 mm × 3 mm I.D., Phenomenex) guard column and a 0.5 μm in-line filter (Idex, Health & Science) were mounted before the MS ion source. Methanol:dichloromethane (1:1, *v/v*)^34,35^ with a flow rate of 0.2 mL min^−1^ was pumped as an elution solvent from the HPLC system (Accela UHPLC 1250 system, Thermo Fisher Scientific). Acetic acid (Merck) in methanol 0.2%, *v/v* was added to the HPTLC effluent in a ratio 1:40, *v/v* prior to the introduction into the MS system^34,35^. Atmospheric-pressure chemical ionisation (APCI) in positive ion mode was used for compounds ionisation. Ion source parameters were set as follows: transfer capillary and vaporiser temperature 300 °C, sheath gas flow rate 30 arbitrary units (a.u.), auxiliary gas 10 a.u. and discharge current 3 μA. MS^n^ experiments were carried out by fragmentation of the target precursor ions using collision energy of 35%^35^.

#### Case-study 4: Quality control of an active pharmaceutical ingredient (API) using UHPLC-ESI-MS

Antidepressant API (confidential) and its related impurities were separated using an Acquity UPLC BEH C18 column (100 mm × 2.1 mm; 1.7 µm, Waters Corporation Milford, MA, USA) connected to a 0.5 μm in-line filter (Idex, Health & Science) using an UHPLC-MS system (Dionex Ultimate 3000 - LCQ Fleet, Thermo Scientific). Mobile phase consisted of 10 mM ammonium bicarbonate buffer pH 10.77 (A):methanol (B) (20:80, *v/v*) under isocratic elution and a flow rate of 0.2 mL min^−1^ was employed. Mobile phases A (see Supplementary Information) and B were filtered through 0.1 µm PTFE membrane filter (Merck Millipore, Carrigtwohill, Ireland). Column and autosampler temperatures were 30 °C and 10 °C, respectively and the run time was 30 min. Total ion current chromatograms (TIC) were acquired in the *m/z* range 100–1,000. Plastic syringe leachables obtained by methanol were analysed with the same method.

#### UHPLC-ESI-MS method for determination of oleamide-leaching labware

Prepared extracts, listed in Table 1 (labelled ‘LC-MS’), were divided into two groups: ones obtained after single and others obtained after multiple labware extractions (see Supplementary Information). Former were prepared without any further pre-treatment, thus simulating labware standard laboratory use, while latter were dried and re-dissolved in 5 mL of methanol. In addition, 5 mL plastic syringe extracts were obtained after 1^st^, 2^nd^, 3^rd^, 4^th^, 5^th^ and 26^th^ rinse with methanol. Water was also tested as an extraction medium of 5 mL plastic syringe. During the extraction procedures, the use of other plastic labware was avoided in order to prevent false positive results for oleamide. All labware methanolic extracts, water extract, methanol and methanol treated with N_2_ flow (prepared to concentrate possible impurities) as blanks were analysed for oleamide by LC-MS.

The analyses of oleamide were performed on an HPLC-MS system in (+)HESI (Dionex Ultimate 3000 - LCQ Fleet). The UHPLC method developed for quality control of API (case-study 4) was applicable for determination of oleamide and is described above. Standard solution of oleamide (25 μg mL^−1^ in methanol) was used to optimise the MS parameters, which were as follows: capillary and heater temperatures 350 °C and 150 °C respectively, sheath gas flow rate 19 a.u., auxiliary gas flow rate 10 a.u., sweep gas flow rate 0 a.u., spray voltage 4.00 kV, capillary voltage 23.41 V and tube lens 91 V. TICs were acquired in the *m/z* range 100–1,000. Selected ion monitoring (SIM) chromatograms of oleamide (*m/z* 282) were also recorded. Collision energy of 30% was used to fragment the precursor ions in MS^n^ experiments.

#### Interference of oleamide and other plastic extractables with bioassay results

For bioassay testing, stock solutions at 100 mg mL^−1^ were prepared by dissolving oleamide standard and dried plastic syringe extract (0.95 mg, obtained after multiple extraction of 5 mL plastic syringes; case-study 1) in 70%_(aq)_ methanol (Honeywell). Stock solutions of Japanese knotweed rhizome ethanolic (70%_(aq)_, Carlo Erba) and acetonic (70%_(aq)_, Honeywell) extracts (see Supplementary Information) were also prepared.

THP-1 monocyte cell line TIB-202 (ATCC) was cultured in RPMI 1640 medium (Thermo Fisher Scientific, Darmstadt, Germany) supplemented with 0.05 mM β-mercaptoethanol (Sigma-Aldrich), 1% penicillin/streptomycin (Thermo Fisher Scientific) and 10% fetal calf serum (FCS; heat-inactivated, Biochrom, Berlin, Germany) in a humidified atmosphere of 5% CO_2_ at 37 °C. 4 × 10^5^ Cells mL^−1^ were seeded in 100 µL medium in a 96-well microtiter plate (Greiner Bio-One, Solingen, Germany) and were allowed to settle for 1 h. The cells were incubated for 2 h at 37 °C with oleamide or extracts in final concentrations of 1 μg mL^−1^, 10 μg mL^−1^ and 100 μg mL^−1^ in cell culture medium or the same amount of vehicle. Afterwards, the TLR4 of the cells was stimulated with lipopolysaccharide (LPS, LPS-EB; from E. coli O111:B4, Invivogen, Toulouse, France) at a final concentration of 50 ng mL^−1^ (4 h at 37 °C). 100 μL of the supernatant of each well was taken for further experiment using ELISA (BD Biosciences)^32^.

Viability of the remaining THP-1 cells was determined using Alamar Blue Assay (Thermo Fisher Scientific) according to manufacturer’s protocol (10% final concentration of Alamar Blue solution). Fluorescence intensity at an excitation wavelength of 560 nm and an emission wavelength of 590 nm was measured with a microplate reader (Synergy Neo, Biotek, Bad Friedrichshall, Germany)^32^.

ELISA was used to determine the IL-8 concentration in the supernatant of pre-treated THP-1 monocytes. The ELISA was performed according to the manufacturer’s protocol with optimised washing buffer volume and 1:2, *v/v* diluted supernatant in assay diluent. A Synergy Neo plate reader (Biotek) was used to measure absorbance at a wavelength of 450 nm and a reference wavelength of 570 nm. The IL-8 concentration of the samples was calculated according to a standard curve (4 parameter logarithmic) and the dilution factor in the program Synergy Neo (Biotek)^32^.

Analysis of variance (ANOVA) was performed to analyse variance between different experimental groups. Unpaired t-test was performed using GraphPad Prism version 5.01 for Windows (GraphPad Software, San Diego, California, USA) to test the statistical significance of the results. P < 0.05 was considered significant^32^.

## Results

### Interference of oleamide with analytical results (Case study 1 – Case study 4)

#### Case-study 1: Multi-dimensional (HP)TLC fractionation of Japanese knotweed rhizome extract, followed by fraction structural elucidation by NMR

NMR spectrum of the fraction isolated from Japanese knotweed rhizome extract^27^ revealed trace amounts of the target analyte (+)-catechin, while showing presence of impurities (visible in the aliphatic part of the spectra).

^1^H NMR spectra of procedural blanks obtained after multiple extractions of PVDF (d = 25 mm) and H-PTFE (d = 8 mm) filters attached to 20 mL plastic syringes, plastic Pasteur pipettes, 50 mL plastic centrifuge vials and methanol (treated with N_2_ flow to concentrate possible impurities) showed that all analysed labware contributed to some impurities in the final plant fraction (peaks mostly observed in the range between δ 0 and 2.5 ppm). However, the most intensive signals of impurities were observed in the ^1^H NMR spectra of extracts of membrane filters attached to plastic syringes. ^1^H NMR spectra of methanol (treated with N_2_ flow), CD_3_OD, glass storage vial extract and glass NMR tube extract did not show presence of impurities.

HPTLC chromatogram obtained by the in-house method for separation of lipid classes (Fig. 1) showed intensive bands of impurities after derivatisation with molybdophosphoric acid reagent (MoP) at *R*_F_ zones of acetone mobile polar lipids (AMPL; *R*_F_ = 0.07 and *R*_F_ = 0.10) for extracts of different membrane filters attached to plastic syringes (tracks 1, 2, 4 and 5, Fig. 1). To test the PVDF material alone, PVDF filter paper extract was analysed and its HPTLC chromatogram showed only negligible amount of impurities in this region (track 3, Fig. 1**)**. Methanol (treated with N_2_ flow) and 20 mL plastic laboratory syringe, which were used for preparation of all tested filter samples, were also analysed. Methanol (treated with N_2_ flow) showed to be relatively pure (track 10, Fig. 1), which was also confirmed by ^1^H NMR analysis, while 20 mL syringe extract contained large amount of impurities (track 6, Fig. 1) and was therefore identified as the main source of contamination. The extract of 5 mL laboratory syringe of the same producer showed only one of the two abundant impurities at the *R*_F_ zone of AMPL (*R*_F_ = 0.10, track 7, Fig. 1). In addition, extracts of centrifuge vials and plastic Pasteur pipettes showed negligible amount of impurities at the *R*_F_ zone of AMPL (tracks 8 and 9, Fig. 1). All tested extracts shown in Fig. 1 with exception of methanol (treated with N_2_ flow) possessed an additional impurity band in the chromatogram in the *R*_F_ region of hydrocarbons (HC; *R*_F_ = 0.85) of unknown identity. The amount of dry residues obtained after multiple extractions of a 5 mL plastic laboratory syringe was visible to the naked eye (0.95 mg, Supplementary Fig. S2). Oleamide was found as a principal impurity and was confirmed by ^1^H NMR analysis of 5 mL syringe extract, obtained after multiple extractions with methanol. Spectra recorded in CDCl_3_ enabled also detection of the amide group hydrogens (broad singlet at δ 5.25 ppm, Supplementary Fig. S3). This signal was not observed in the ^1^H NMR spectra of the previously analysed extracts acquired in CD_3_OD, due to proton exchange.

The HPTLC separation of lipid classes was further combined with (+)ESI-MS^n^ for additional analyses of extracts of plastic syringes (5 mL and 20 mL, both of the same producer), obtained after multiple extractions. The significant impurity bands at *R*_F_ = 0.10, present in tracks of both plastic syringes, corresponded to the *R*_F_ of oleamide standard (Supplementary Fig. S1). The presence of oleamide in these extracts was further confirmed by MS and MS^n^ (Table 2). The compounds comprising the band at *R*_F_ = 0.07 in the track of 20 mL plastic syringe (Supplementary Fig. S1) were tentatively identified as oxidation products of oleamide (Table 2).

**Table 2:** Mass spectra signals obtained with HPTLC-(+)ESI-MS^n^ analysis of the HPTLC zones in the tracks of 5 mL and 20 mL plastic syringe extracts (Figure S1).

*R*_F_	MS (*m/z*)	MS^2^ (*m/z*)	MS^3^ (*m/z*)
0.07	296 [M + H]^+^	[296]: 279	[296 → 279]: 261, 243
0.07	280 [M + H]^+^	[280]: 263, 245	[280 → 263]: 245
0.07			[280 → 245]: 79−177 (Δ = 14), 77-217 (Δ = 14)
0.07	328 [M + H]^+^	[328]: 296	[328 → 296]: 279
0.10	282 [M + H]^+^	[282]: 247, 265	[282 → 265]: 247
0.10			[282 → 247]: 81−179 (Δ = 14), 79−219 (Δ = 14)

#### Case-study 2: HPLC fractionation of cinnamon extract and identification of the unknown analytes by direct injection (DI)-ESI-MS

**Figure 2:**
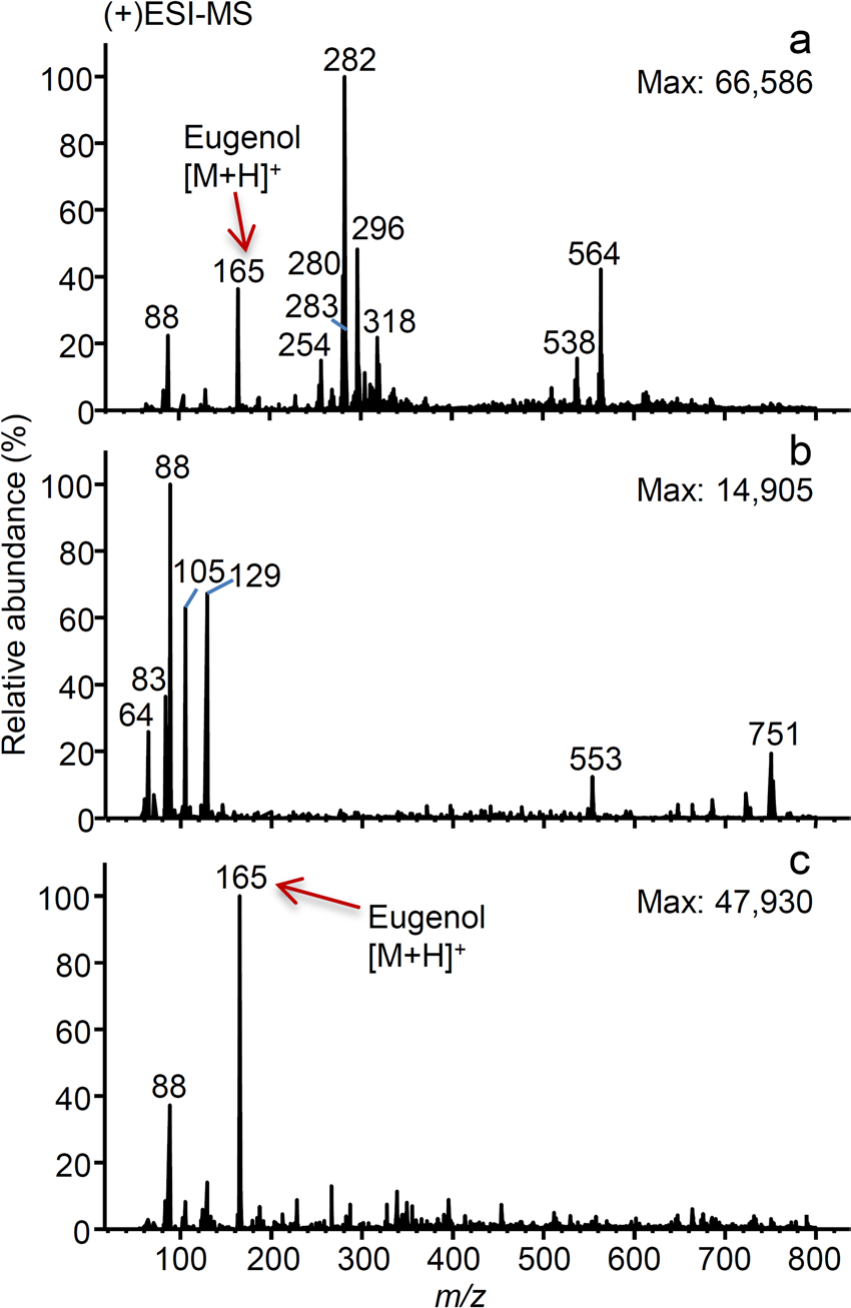
DI-ESI-MS spectra in positive mode, corresponding to: eugenol ethanolic standard solution analysed after filtration (**a**), ethanol as blank, analysed without filtration (**b**) and eugenol ethanolic standard solution filtered using centrifuge tubes containing cellulose acetate membrane filter (**c**).

In the scope of HPLC fractionation of cinnamon (*Cinnamomum verum*) extract and identification of chemical constituents in the potent anti-inflammatory fractions^32^, identification of the compounds was at first hindered by the presence of some contaminant mass peaks. Upon injection of filtered ethanolic eugenol standard (suspected to be present in one of the active extract fractions) into the (+)ESI-MS, mass peaks at *m/z* 282, 280, 296, 318 and 320 appeared, while the mass peak corresponding to eugenol standard at *m/z* 165 was barely visible. Although MS method was further optimised using eugenol standard, contaminant mass peaks at *m/z* 282, 280, 296, 318 and 564 were still dominant (Fig. 2a). In contrast, blank (ethanol) injected without filtration did not show any of these contaminant mass peaks (Fig. 2b). Most of these contaminant peaks were present in MS spectra of the eugenol standard, fractions and blanks, after their filtration, regardless of the solvent type (ethanol, acetonitrile or mobile phase) used for solvation, dilution or concentration and of the type of the filter (PVDF, PTFE) used for filtration. Finally, an MS spectrum obtained for the extract of the plastic syringe, used for filtration, showed that the syringe itself was the main source of these contaminants (*m/z* 282, 296, 564).

Therefore, each plastic and non-plastic labware, used during the bioassay-guided fractionation^32^, was separately tested for leachables using mobile phase as a contact solvent, unless stated otherwise. Both PVDF and PTFE filters mounted to a glass gas tight syringe were extracted and tested apart from the plastic syringe. PTFE filters gave slightly lower MS background in comparison to PVDF, when tested together with leachables-free glass gas tight syringe. Moreover, no significant difference was observed between both filter types after filtration of ethanolic eugenol standard with or without pre-rinsing with ethanol. Glass fraction collector tubes, glass syringe, plastic micropipette tips, plastic 15 mL centrifuge vials, used for fraction storage, and centrifuge tubes containing cellulose acetate membrane filter did not cause any contamination problems in MS. Plastic Pasteur pipettes leached contaminants at *m/z* 282, 338 and 360. Two additional types of sterile plastic syringes extracted with mobile phase or ethanol showed similar profiles as the previously tested. After extraction with the mobile phase, glass Pasteur pipettes showed MS spectrum with multiple mass peaks of lower intensity. Data on the oleamide-leaching labware, tested by the DI-MS method are summarised in Table 1. Finally, centrifuge tubes containing cellulose acetate membrane filter enabled clear MS background with no interferences for eugenol standard in ethanol (base peak, Fig. 2c).

#### Case study 3: HPTLC-APCI-MS analysis of Japanese knotweed leaves extract

**Figure 3:**
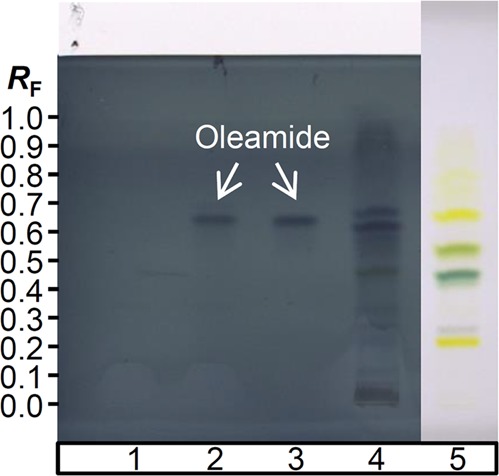
Chromatograms of: blank (90% acetone_(aq)_:1 M TEAA with pH 7; 85:15, *v/v*) without filtration (**1**), filtered blank (**2**), oleamide methanolic standard solution (**3**), and filtered Japanese knotweed leaves extract (**4, 5**). Solutions were applied as 8 mm bands on a pre-developed C_18_ RP HPTLC plate and developed by 0.1% TBHQ in methanol:acetone (1:1, *v/v*)^34^. Plate was visualised under white light illumination without (track **5**) and with post-chromatographic derivatisation with MoP (tracks **1–4**).

Identification of the unknown analytes extracted from Japanese knotweed leaves observed in the HPTLC chromatogram was performed in combination with (+)APCI-MS^n^. MS spectra of a zone of interest at *R*_F_ = 0.66 showed presence of precursor mass peaks at *m/z* 282 (MS) and product mass peaks at *m/z* 265, 247 (MS^2^) and *m/z* 247 (MS^3^), suggesting the presence of oleamide as interference. Therefore, an HPTLC plate with applied non filtered and filtered blank, oleamide standard solution and filtered Japanese knotweed leaves extract (in duplicate) was developed according to our method for carotenoids^34^ and derivatised (except the track of the last application) by MoP (Fig. 3). Filtered blank and filtered Japanese knotweed extract showed bands at the *R*_F_ of oleamide standard (0.64, Fig. 3).

#### Case-study 4: Quality control of an active pharmaceutical ingredient (API) using UHPLC-ESI-MS

**Figure 4:**
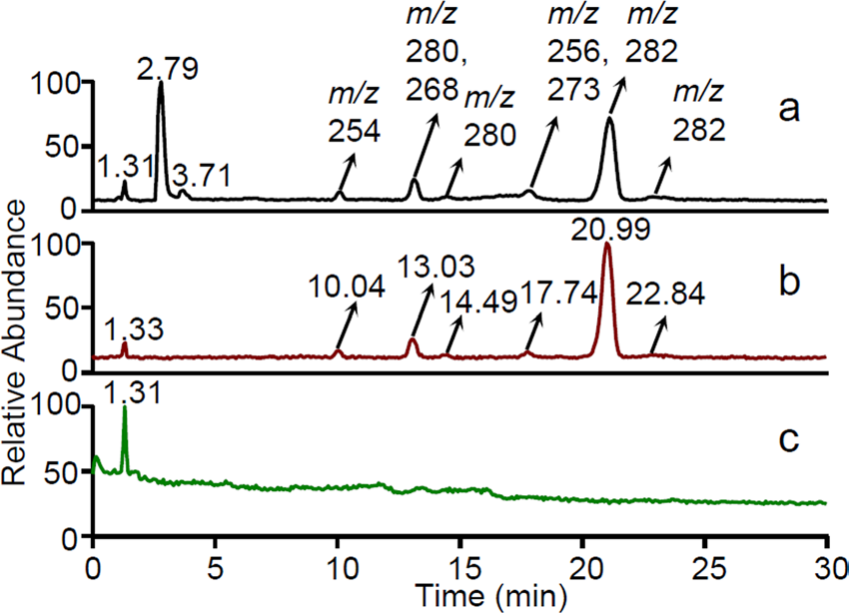
TIC chromatograms of: filtered API solution (**a**), methanolic extract of 5 mL plastic syringe (**b**) and blank (**c**).

An UHPLC-MS method was developed to separate antidepressant API and its related substances. Besides the peaks of API and the key impurity, both eluting in less than 5 min, few other peaks were observed at *t*_*R*_ 10.04, 13.03, 14.49, 17.74, 20.99 and 22.84 min, which were later also found in the TIC chromatograms of filtered API solution and methanolic extract of a 5 mL plastic syringe (Fig. 4).

#### UHPLC-ESI-MS method for determination of oleamide-leaching labware

The method developed to separate API and its related substances (case-study 4) was adopted for identification of oleamide-leaching labware. MS method was optimised for oleamide standard employing (+)HESI. By fragmentation of the precursor ion (*m/z* 282), mass peaks at *m/z* 265 and 247 were obtained. Fragmentation of the precursor ion at *m/z* 265 resulted in product ion at *m/z* 247, while by its further fragmentation, two sets of cluster ions with mass difference of 14 (*m/z* 81–179 and *m/z* 79–219) were observed. It is evident from Table 1, that oleamide migrates into the extraction media (which is methanol or water in case of 5 mL plastic syringe) from some plastic labware and surprisingly also from some non-plastic labware, such as a certain type of HPLC glass vial inserts, either after single or multiple extraction procedures. The area of oleamide peak in the extract chromatograms of a 5 mL plastic syringe obtained after 1^st^, 2^nd^, 3^rd^, 4^th^ and 5^th^ rinse with methanol was plotted against the number of the consecutive rinsing steps (Supplementary Fig. S4). Area and consequently amount of extracted oleamide decreased with increasing the number of consecutive rinses.

Other contaminants’ peaks were also observed in the LC-MS chromatograms of the analysed labware extracts and their identity was tentatively determined by the help of the literature data (Supplementary Table S1).

#### Interference of oleamide and other plastic extractables with bioassay results

THP-1 monocyte cell line was incubated with 1 μg mL^−1^, 10 μg mL^−1^ and 100 μg mL^−1^ oleamide solution and plastic syringe extract (for which presence of oleamide was confirmed), followed by stimulation with LPS to analyse the effect on the TLR4 signaling pathways *in vitro*. The viability of LPS-stimulated cells treated with oleamide, syringe extract and vehicle (70% methanol) was comparable to those of untreated cells (Supplementary Fig. S5a). A small but significant decrease of IL-8 secretion was observed in THP-1 monocytes pre-treated with 100 μg mL^−1^ oleamide solution, whereas the same concentration of plastic syringe extract led to a significant increase of IL-8 secretion (p < 0.0001; Supplementary Fig. S5b). The same tendency could be observed when normalizing the IL-8 secretion to the viability of the cells (p < 0.001; Supplementary Fig. S5c).

## Discussion

Four case-studies with unrelated topics have been performed independently (in the course of different projects, by different researchers, and some of them even in different laboratories), initially without insight into the presence of oleamide leachate. Its leaching was then confirmed by subsequent analyses after realising that oleamide may present a contaminant. Careful experiments have been performed for each case-study separately to exclude the possibility that oleamide is part of the sample chemistry. Each case-study was carried out using different analytical techniques, which complementarily demonstrated presence of oleamide as a contaminant. The fact that oleamide interfered with all shown results aims to raise the awareness of the researchers during execution of their experiments. Some strategies elaborated in this study may serve as future guide on how to exclude the potential interferences of oleamide and other related leachables and extractables.

In case-study 1, ^1^H NMR spectra of extracts of different laboratory materials were recorded, since ^1^H NMR spectrum of the isolated fraction of Japanese knotweed rhizome extract showed presence of impurities. Oleamide, which was specified as a plunger lubricant by the manufacturer of 5 and 20 mL syringes, was confirmed as a principal impurity by ^1^H NMR analysis of 5 mL syringe extract, obtained after multiple extractions with methanol. Presence of oleamide in the extract was additionally supported by the ^1^H NMR data published elsewhere in the literature^5,13,19,36^. The applicability of the in-house HPTLC method, which was reported to be capable for separation of representative compounds belonging to 9 different lipid classes: L-α phosphatidylcholine (phospholipids, PL), α-monopalmitin (acetone-mobile polar lipids, AMPL), cholesterol (sterols, ST), cetyl alcohol (fatty alcohols, FA), palmitic acid (free fatty acids, FFA), tripalmitin (triacylglycerols, TAG), 3-hexadecanone (ketones, KE), stearyl palmitate (wax esters, WE), and squalane (hydrocarbons, HC)^28^, was proven useful for separation and detection of extracted impurities originating from labware. Chromatographic methods for separation of compounds according to lipid classes were already described as important in the analysis of plastic additives^37^. Molybdophosphoric acid reagent (MoP) showed to be an appropriate derivatisation reagent for quick detection of oleamide and other impurities originating from laboratory materials. Plastic syringe (5 mL and 20 mL) extracts were further chosen for HPTLC-MS^n^ analysis, due to the high intensity of their impurity bands observed in HPTLC chromatogram (Fig. 1**)**, as well as the abundance of their impurity signals in ^1^H NMR spectra. After fragmentation of the ions at *m/z* 282, 280 and 296, peaks in MS^2^ spectra showed loss of ammonia (−17 *m/z*) and water (−18 *m/z*), that are typical for monounsaturated primary fatty acid amides^38^. Further fragmentation led to clusters with mass difference of the fragments of 14 *m/z* (Table 2), which correspond to loss of -CH_2_- groups, typical for acyl chain lipids^38^. The issues with leachables from laboratory materials, interfering with ^1^H NMR analysis of the Japanese knotweed isolate, were solved after excluding the contaminants-leaching labware^27^.

Identification of the compounds present in the potent anti-inflammatory fractions of *Cinnamomum verum* extract (case-study 2), using DI-(ESI)-MS^n^, was troubled by the presence of contaminant mass peaks, which appeared also in the MS spectra of the standard and blanks exposed to filtration, regardless the solvent or filter type used. From the observed mass peaks (*m/z* 282, 280 and 296), the peak at *m/z* 282 was identified as oleamide, while others were tentatively assigned to its oxidation products (Table 2). The observed ion with *m/z* 318 may correspond to bisphenol A monobenzyl ether^39^ or dihydroxy oleamide^40^. To rule out the source of contamination, procedural blanks were prepared and analysed by MS. MS spectrum of plastic syringe extract showed that the syringe itself was the main source of these contaminants (*m/z* 282, 296, 564). Contaminants observed in the MS spectrum of the plastic Pasteur pipettes leachate could be tentatively identified as oleamide (*m/z* 282) and erucamide (*m/z* 338), while possible adduct of the latter with sodium was also observed (*m/z* 360). Therefore, the use of glass syringes is recommended over the use of plastic Pasteur pipettes and sterile plastic syringes, and the use of plastic micropipette tips, which extract did not show contaminant signals in MS, is encouraged over glass Pasteur pipettes (multiple mass peaks of lower intensity). Moreover, centrifuge tubes containing cellulose acetate membrane filter, used in this study, showed to be an appropriate substitute for the plastic filtration set.

In the case-study 3, an observed MS spectrum was initially assumed to correspond to a Japanese knotweed extract compound. However, procedural blank (filtered blank) applied on the HPTLC plate showed that the compound did not originate from the plant extract itself, thus proving that oleamide leached from the used filtration set. Oleamide was additionally confirmed by (+)APCI-MS^n^ and fragmentation patterns were matching with those obtained by (+)ESI-MS^n^ analysis of oleamide band (*m/z* 282, Table 2). The band observed in the plant extract chromatogram, corresponding to *R*_F_ of oleamide (track 4, Fig. 3), was slightly shifted towards higher *R*_F_ in comparison to the standard’s band, most probably due to the influence of the complex plant matrix. As expected, oleamide band was not observed on the underivatized part of the plate under white light illumination (track 5, Fig. 3). However, its interference with coeluting bands of carotenoids lutein and zeaxanthin was obvious after plate derivatisation with MoP and was confirmed by HPTLC-MS analysis.

Many signals, detected by UHPLC-MS in case-study 4, were initially considered to be related impurities of API. However, by comparing TIC chromatograms of filtered API solution and methanolic extract of a 5 mL plastic syringe (Fig. 4), laboratory plastic syringe was found as the main source of contaminants. Other chromatographic peaks, showing *m/z* at 254, 256, 268, 273, 280, 282, were present in the procedural blank chromatogram (Fig. 4b), which potential identity (e.g. palmitamide at *m/z* 256, elaidamide at *m/z* 282, compounds related to oleamide standard or other) might be revealed by the data summarized in Supplementary Table S1.

Oleamide was detected as the main plastic labware migrant and analytical interference through the four case studies (case-studies 1–4) elaborated above. Its leaching from labware was proven by ^1^H NMR, HPTLC, HPTLC-ESI-MS (case-study 1), DI-ESI-MS (case-study 2), HPTLC-APCI-MS (case-study 3) and UHPLC-ESI-MS (case-study 4) methods. Through these four case studies, oleamide was presented as major contaminant and interference, which if not carefully considered, could lead to false-positive analytical results during execution of phytochemical (case-studies 1–3) or pharmaceutical (case-study 4) studies, especially using the untargeted approach. Different labware items were tested for leaching of oleamide using ^1^H NMR (case-study 1), HPTLC (case-study 1) and DI-MS (case-study 2) methods.

To prevent future misleading oleamide-related results, an UHPLC-ESI-MS method was developed for identification of oleamide-leaching labware. Using C18 column and isocratic elution of the mobile phase, a good selectivity for oleamide and other accompanying leachables and extractables, present in the procedural blanks, was provided. Although oleamide is not detectable under all analytical conditions (e.g. on underivatized part of the HPTLC plate in case-study 3), the employed mobile phase (80% methanol, 20% ammonium bicarbonate buffer, pH 10.77) and the optimized MS tune enabled its ionisation in the ion source and subsequently its detection. MS and MS^n^ spectra of oleamide, obtained upon UHPLC analysis, showed the same mass signals (*m/z* 282 in MS and *m/z* 265 and 247 in MS^2^) as presented in Table 2 and discussed above. Different labware was tested by this method and the results summarized in Table 1 under the label ‘LC-MS’ show that oleamide migrates into the extraction media (mostly methanol) from some plastic labware and surprisingly also from some non-plastic labware either after single or multiple extraction procedures. The presence of oleamide in a certain type of HPLC glass vial inserts, used for analysis of extracts, was observed. Some glass vial inserts are likely coated with oleamide, allowing easy liquid filling. The other glass vial insert type, which was proven to be oleamide-free (Table 1), was therefore further used for extracts analyses. Since oleamide was present in the methanolic extract of a 5 mL laboratory plastic syringe, which is also available on the market for parenteral administration, water as a pharmaceutical formulation simulant was used to prepare an additional extract (Table 1). To summarize the results from Table 1, 13 out of 28 tested labware proved positive for leaching of oleamide. This observation was however not connected to any specific brand (20 tested) as different products from the same brand showed opposite results in some cases. In this context, it is also important to point out that different batches of the same labware products may show different results. It is highly probable, that labware manufacturers change the supplier of the plastic materials from time to time. Due to the above reasons, brands of the labware used in this study are kept confidential.

Washing of oleamide from 5 mL plastic syringe was tested by performing consecutive rinsings with methanol (Supplementary Fig. S4). Significant difference among peak areas of oleamide was observed between the first and the second rinse, meaning that the largest amount of oleamide can be washed away if a pre-cleaning step is included into the sample preparation procedure. Every next analysed syringe rinse (from 2^nd^ to 5^th^) revealed comparable extracted amount of oleamide. Even 25^th^ rinsing with methanol could not completely rinse out all oleamide from the plastic syringe (Table 1). This observation is reasonable, since as a lubricant, oleamide is intended to constantly diffuse from the polymer to its surface^41^.

Other contaminants, observed in the LC-MS chromatograms of the analysed labware extracts, among which the commonly occurring ones were tentatively identified, are listed in Supplementary Table S1. Some of them are plastic additives with the function to improve the polymer functionality, performance and aging properties. According to their role, plastic additives can be divided to plasticizers, antioxidants, flame retandants, acid scavengers, lubricants, antistatic agents, pigments, heat, light and thermal stabilisers^1^. In addition, some non intentionally added substances could also be found as leachables and extractables. They may originate from the starting polymer material or may appear as intermediates, decomposition or reaction products during polymerisation or thermal processing of the polymers^41^. Although few other compounds besides oleamide, listed in Supplementary Table S1, already appeared through the four-case studies (e.g. erucamide, oxidation products of oleamide, bisphenol derivatives etc.) and could also represent analytical interference especially when untargeted analyses are executed, their detailed studying was out of the scope of this manuscript and might be part of another study. Some plastic migrants are known and are sometimes even declared in the technical specifications of certain products. However, a huge number of leachables and extractables remains unidentified and their characterization would be of future great importance^41^.

In the last part of this study, oleamide and a plastic syringe extract, which showed to contain oleamide, appeared to interfere with the current (inflammatory) bioassay as they exhibited activity. Several biological studies have already reported anti-inflammatory activity of oleamide^9–13^ in concentration of 100 μg mL^−1^ ^12^. In our employed bioassay system, oleamide showed small but significant (p < 0.001) anti-inflammatory activity in high doses (100 μg mL^−1^). Interestingly, methanolic extract of a 5 mL laboratory plastic syringe showed pro-inflammatory activity in concentration of 100 μg mL^−1^ (p < 0.001). Presence of other impurities leaching from the laboratory plastic syringe, as detected by UHPLC-ESI-MS, may contribute to this effect. Many impurities, leached from 5 mL plastic syringe, were assigned as oleamide standard related compounds (Supplementary Table S1). Although oleamide itself, found as major compound of a 5 mL plastic syringe (case-study 1), showed slightly anti-inflammatory activity in high doses, its combination with other impurities exhibited considerable opposite effect. It is not clear whether this activity is attributed to some of the extracted impurities or certain combination thereof. Interferences of oleamide along with some other plasticware leachables with other bioassays have already been reported^8,15,17^. Consequently, filtration of samples using filters attached to plastic syringes, could contribute to misleading results in the case vehicles are not handled the same way. The sample handling procedure using contaminants-leaching labware (e.g. sample filtration through filters attached to plastic syringes) should either be avoided or carefully executed for the purposes of bioassay screening and must be repeated for the vehicle as well. Batch-to-batch variation of labware items should also be taken into account. Therefore, the reliability of the already published bioassay studies, in which plastic labware has been part of the experiment, is questioned. As an example of such questionable results, an in-house screening study of anti-/pro-inflammatory activity of filtered 70%_(aq)_ ethanolic and 70%_(aq)_ acetonic extracts of Japanese knotweed, tested with the same biological assay, is presented (Supplementary Fig. S6). According to the experiment, both extracts in concentration 100 μg mL^−1^ showed pro-inflammatory activity (Supplementary Fig. S6). Since all extracts were filtered through a plastic syringe before the biological testing and having in mind the pro-inflammatory activity of the methanolic extract of a plastic syringe in concentration of 100 μg mL^−1^ (Supplementary Fig. S5), the reliability of the obtained results is questionable. Therefore, the extraction of the plant material and its further handling should be repeated either by exclusion of all interfering laboratory materials or by filtering the vehicle as well, to prove the bioactivity of the plant extracts themselves.

To summarize, by the current study oleamide was proven to interfere with analytical or bioassay results. Its leaching from different labware was also confirmed and was not found to be related to a certain brand. Therefore, the question, whether oleamide elaborated by the scientific literature is real analyte or just interference, should be placed. Many publications report oleamide in various samples, predominantly of natural origin. Oleamide, on the other hand is also a natural compound, which was found in the cerebrospinal fluid of sleep-deprived animals^5^. It was isolated from green algae *Codium fragile*^11^ and *Arctium lappa*^12^ extracts, which showed potent anti-inflammatory activity. It was also identified in the extract of a green alga *Rhizoclonium hieroglyphicum*^18^. However, it is not clear if authors were aware of oleamide problem or most importantly if they overlooked its presence as a contaminant. Oleamide and other fatty acid amides (myristamide, palmitamide, linoleamide, elaidamide, stearamide and erucamide) were reported as main compounds contributing to the toxicity of a harmful alga *Prymnesium parvum*^19^. It may or may not be a coincidence that all these fatty acids are also used as lubricating agents in polymer industry^26^. Specific individual cases, where analytical results were erroneously interpreted due to plastic contamination, were already mentioned. Namely, a study identified oleamide as a predominant compound of the human meibum^20^. However, this finding was already rejected by a subsequent study, where oleamide was marked as plastic contamination of the human meibum samples^22^. Oleamide was also found as contaminant in geochemical samples and according to the authors of the study, has been introduced along with some other contaminants during sampling or subsequent samples handling^23^. A study has identified oleamide in silk extract of spider *Pholcus beijingensis*^21^, however authors of a later study, who also found oleamide as contaminant in some ant cuticle samples have disagreed and verified that oleamide is originating from the laboratory plasticware^24^. Oleamide was also reported in some archaeological vessels samples, which enabled authors, although aware of possible contamination, to connect its presence to vessels content of vegetal origin^25^. Recently, oleamide was detected in 2 out of 7 commercial *Stevia rebaudiana* (Bert.) Bertoni extracts^42^ and in *Cyperus esculentus* rhizome extract^43^ although its plant origin have not been proven. Oleamide could have been involved in the analysed extracts during extraction procedures. The authors of the latter study^43^ have stated to be aware about the presence of oleamide in plastics and avoided contacts with plastic labware during their experimental work, though testing of procedural blanks is not mentioned. However, leaching of oleamide from some non-plastic materials (proven by the current study) is something new to the professional public and should also be taken into consideration in the future.

Therefore, all published results dealing with oleamide should be carefully considered, as it can in fact represent a contaminant introduced in the sample through sample preparation methods^44^ which employ plastic or other labware (case-studies 1–4 and Table 1). Having this contamination in mind, a question regarding the reliability of the already published oleamide related studies is raised.

The dual origin of oleamide (industrial lubricant and natural compound) makes its differentiation (analyte/interference) extremely difficult and only carefully and well-designed experiments may help to rule out any possible contamination.

## Conclusions

Through four unrelated analytical (phytochemical and pharmaceutical) case-studies and employing different analytical techniques (HPTLC, HPTLC-ESI-MS, HPTLC-APCI-MS, DI-ESI-MS, UHPLC-ESI-MS and ^1^H NMR), plastic labware was found to leach oleamide into the contact solvents, recognised as compatible with the polymer. Surprisingly, some non-plastic labware, such as glass vial inserts, were also found as source of oleamide. In 13 out of 28 tested labware item types, commonly used during sample handling, the presence of oleamide was confirmed. Analysis of the labware items, purchased from 20 different manufacturers, demonstrated that leaching of oleamide was not related to a certain brand. Due to oleamide interferences, careful interpretation of the case-studies results was needed.

This clearly indicates the possibility of reporting false positive analytical results, especially when taking into account the fact that oleamide can be found in nature as well. Many publications report oleamide as bioactive compound isolated from natural sources, which can in fact be a lubricant interference leached from the used labware.

Due to the measured anti-inflammatory effect of oleamide and the pro-inflammatory effect of the plastic syringe extract in a TLR4-based bioassay, handling of a sample with contaminants-leaching labware (e.g. filtration) could represent a likely source of error in this bioassay. Although similar observations were reported for other bioassays, the effect on this bioassay is considered for the first time.

Therefore, the reliability of the already published analytical and bioassay studies, performed using oleamide-leaching labware, should be questioned.

This study aims to raise the awareness regarding oleamide-leaching labware and improve the quality of the published results in the future. To prevent further misinterpretation of analytical and bioassay oleamide-related data, few general strategies are proposed. In case of suspected oleamide interferences, one should test and thus avoid using oleamide-leaching labware. For instance, if filtration is needed, an oleamide-free labware suitable for filtration (e.g. glass tight syringe with mounted leachables-free filter type or pre-tested centrifuge tubes with cellulose acetate membranes) can be employed. If the use of oleamide-leaching labware cannot be avoided, preparation of exact procedural blanks, by simulating each step of the sample preparation procedure, is recommended. Finally, when chromatographic fractionation is included in the experiment, blanks can be prepared by fraction collection of blank chromatographic (e.g. HPLC, (HP)TLC) runs. For straightforward and unequivocal identification of oleamide from oleamide-leaching labware, *de novo* developed UHPLC-ESI-MS method, besides other methods used in the case-studies, is proposed.

## Supplementary information

Supplementary information.

## Data Availability

All data generated or analysed during this study are included in this published article and its Supplementary Information Files.
