# Supplementary material for: Interference of oleamide with analytical and bioassay results
Source: Sci Rep. 2020 Feb 7;10:2163. doi: 10.1038/s41598-020-59093-1 (PMC7005802; doi:10.1038/s41598-020-59093-1)
Supplement: Supplementary file 1 — Supplementary information. [file 41598_2020_59093_MOESM1_ESM.pdf]

SUPPLEMENTARY INFORMATION:

Interference of oleamide with analytical and bioassay results

Urška Jug[a,b,‡], Katerina Naumoska[a,c,‡], Valentina Metličar[a,b], Anne Schink[c], Damjan Makuc[d], Irena Vovk[a,*], Janez Plavec[b,d,e], Kurt Lucas[c]

[a]Department of Food Chemistry, National Institute of Chemistry, Hajdrihova 19, 1001 Ljubljana, Slovenia

[b]Faculty of Chemistry and Chemical Technology, University of Ljubljana, Večna pot 113, 1000 Ljubljana, Slovenia

[c]Multiphase Chemistry Department, Max Planck Institute for Chemistry, Hahn-Meitner-Weg 1, 55128 Mainz, Germany

[d]Slovenian NMR Centre, National Institute of Chemistry, Hajdrihova 19, 1001 Ljubljana, Slovenia

[e]EN-FIST Centre of Excellence, Trg Osvobodilne fronte 13, 1000 Ljubljana, Slovenia

[‡]*These authors contributed equally.*

**Corresponding author:*
Tel.: +386 1 4760 341;
E-mail address: irena.vovk@ki.si

▪ MATERIALS AND METHODS

Case-study 1:

Impurities from different laboratory materials were extracted using pool of methanol (LC-MS grade, Honeywell Reagents) contained in a glass beaker. Four different types of membrane filters for syringe were extracted separately as follows: i) polyvinylidene fluoride (PVDF, 0.45 μm, d=25 mm), ii) PVDF (0.45 μm, d=8 mm), iii) hydrophilic polytetrafluoroethylene (H-PTFE, 0.20 μm, d=8 mm) and iv) regenerated cellulose (RC, 0.45 μm, d=25 mm). Methanol (20 mL from the pool of 40 mL in total) was pushed five times through five pieces of filter attached to a plastic syringe (20 mL, one per each filter type). Five items of a membrane filter paper (PVDF type GV, 0.22 μm) cut in pieces were extracted in 40 mL methanol while stirring with a magnetic stirrer for 15 min. Methanol (20 mL (i) or 5 mL (ii) from the pool of 40 mL in total) was also pushed through plastic laboratory syringes in the following manner: i) one 20 mL syringe was rinsed 25 times; (ii) four 5 mL syringes were rinsed 25 times. A 50 mL centrifuge vial was filled with 40 mL of methanol, ultrasonicated for one hour and the extract was transferred to another 50 mL centrifuge vial where the procedure was repeated. Five pieces of one plastic Pasteur pipette type were rinsed 15 times each with 2 mL from the pool of 40 mL of methanol. Amber glass storage vial was filled with 40 mL of methanol.

Case-study 3:

Fresh leaves of Japanese knotweed were frozen by liquid nitrogen, lyophilised for 24 h at -50 °C (Micro Modulyo IMAEdwards, Bologna, Italy) and pulverised for 1 min at 1,700 min^{-1} (Mikro-Dismembrator S, Sartorius, Gottingen, Germany). Pulverised samples (20–30 mg) were further weighed into 45 mL glass Carousel tubes to which 10 mL of solvent mixture composed of 90% $acetone_{(aq)}$ and 1 M triethylammonium acetate (TEAA) (pH 7)[33] (both Honeywell) in ratio of 85:15, *v/v* was added. Extraction was executed by Carousel 12 Plus apparatus (Radleys, Safron Walden, UK) in dark environment, under N_2 atmosphere and using magnetic stirring at room temperature for 15 min. The obtained sample extracts were subsequently centrifuged at 4,200 rpm for 5 min and supernatants were filtered through a membrane filter.

Case-study 4:

Mobile phase A was prepared by dissolving ammonium bicarbonate (0.79 g, Honeywell) in 950 mL Milli-Q water (18 MΩ cm, Millipore, Bedford, MA, USA) and adding ammonia solution (32%, Sigma-Aldrich) to obtain pH of 10.77. Milli-Q water was added to the final solution to obtain 1 L of buffer.

UHPLC-ESI-MS:

Extracts, obtained by single extraction of a labware with methanol, were prepared using the following procedure: 1,000 μL plastic pipette tips were rinsed with equal volume of methanol; 15 mL and 50 mL plastic centrifuge vials were filled with respective volume of methanol and ultrasonicated for 15 min; plastic Pasteur pipettes were rinsed with cca. 2 mL methanol; 2 mL microcentrifuge tubes without and with cellulose acetate filter were filled with 2 mL and 600 μL methanol, respectively and centrifuged at 13.4 rpm for 5 min; H-PTFE (0.2 μm) and PVDF (0.45 μm) syringe filters were exposed to 10 mL of methanol, which was pushed through using a 10 mL glass syringe; 5 mL plastic syringe was rinsed with the same volume of methanol.

Among the previously prepared samples for HPTLC analysis (case-study 1), extracts obtained after multiple extractions of 5 mL and 20 mL plastic syringe, 50 mL plastic centrifuge vial, plastic Pasteur pipettes and methanol treated with N_2 flow, were used. In addition, few new extracts were obtained as follows: 5 pieces of 1,000 μL pipette tips were rinsed with equal volume of methanol in 15 repetitions each; 10 pieces of 2 mL microcentrifuge tubes were filled with 2 mL of methanol and vortexed at 2,800 rpm for 2 min; 10 mL of methanol (from a pool of 40 mL in total) was pushed 5 times through 5 pieces of PVDF syringe filter (0.45 μm) attached to a 10 mL glass syringe.

Other non plastic laboratory materials were examined after single extraction of a labware as well: two glass vial inserts obtained from different manufacturer, 1 mL and 5 mL glass syringes with needle and glass Pasteur pipette, were rinsed with methanol in the largest possible volumes. Part of a laboratory glove was cut into pieces, dipped into 20 mL methanol and the extract was concentrated 10 fold.

Bioassay:

Japanese knotweed rhizomes were liophilised and pulverised and the obtained material was suspended in $70\%_{(aq)}$ ethanol (Carlo Erba) and $70\%_{(aq)}$ acetone (Honeywell), separately thus obtaining 750 mg dry extract in 5 mL solvent. The samples were further vortexed for 1 min, ultrasonicated for 15 min, centrifuged for 5 min at 4,200 rpm and filtered through PVDF filter mounted to a 20 mL plastic syringe.

■ **SUPPLEMENTARY FIGURES S1–S5 AND SUPPLEMENTARY TABLE S1**

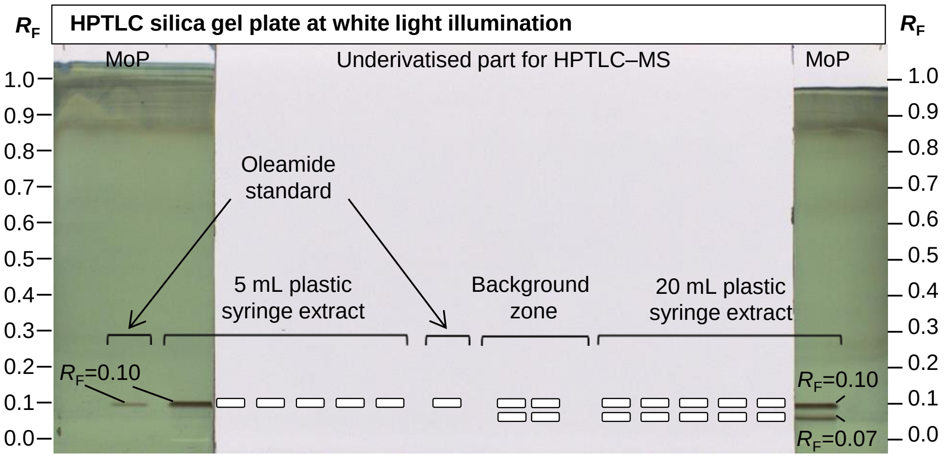

Figure S1. Chromatograms of methanolic extracts of 5 mL and 20 mL plastic syringes and of oleamide standard solution. Plastic syringes extracts (50 mm bands) and oleamide standard solution (8 mm bands) were applied on twice pre-developed HPTLC silica gel plate and developed using HPTLC method for lipid classes. The bands of impurities at R_F = 0.07 (track of 20 mL plastic syringe extract) and R_F = 0.10 (tracks of both 5 mL and 20 mL plastic syringe extracts) were visualised after post-chromatographic derivatization of left and right plate sides with MoP. Eluted zones in the non-derivatised part of the plate by HPTLC-MS interface are schematically represented by white rectangles.

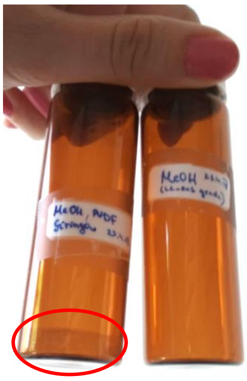

Figure S2. Dry residues, visible to the naked eye, obtained after multiple filtrations of methanol through PVDF filter (d=25 mm), mounted to a 20 mL plastic syringe, and subsequent evaporation of the solvent (left vial), and absence of dry residues (not visible to the naked eye), where the same amount of methanol was only evaporated, avoiding the filtration step (right vial).

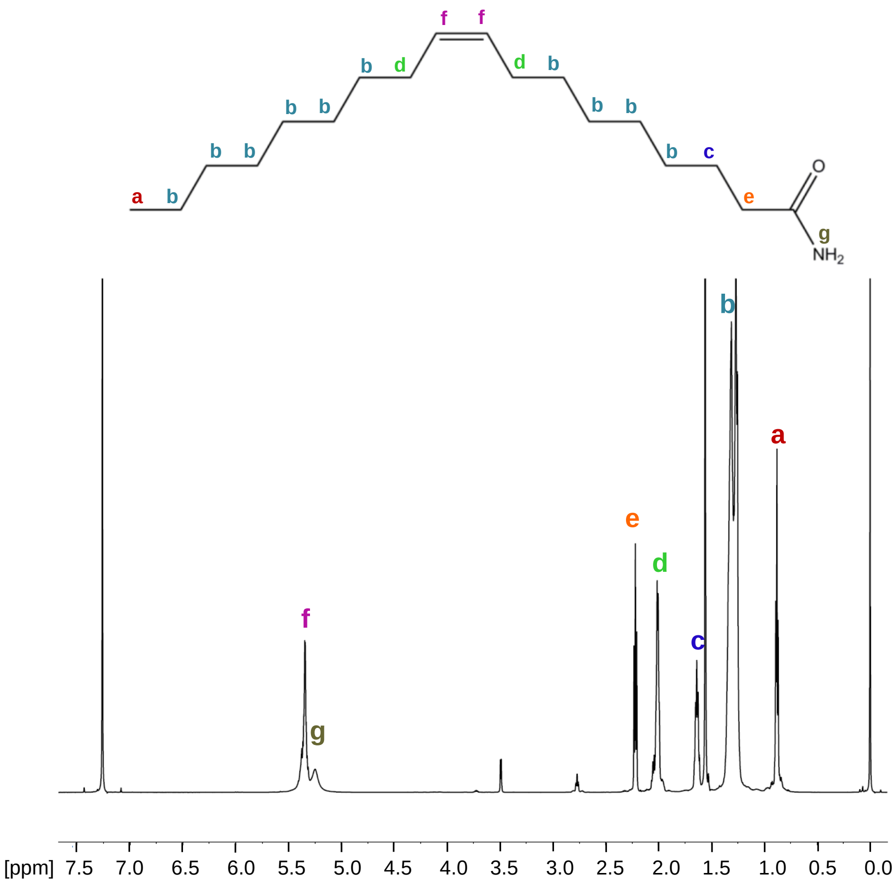

Figure S3. ^{1}H NMR (600 MHz, cca. 950 µg of dry 5 mL plastic syringe extract dissolved in 0.5 mL of $CDCl_3$): δ (ppm) 5.35 (m, 2H, olefinic protons), 5.25 (s, 2H, amide protons), 2.22 (t, *J*=7.7 Hz, 2H, α-$\boldsymbol{H_2}$), 2.02 (m, 4H, allylic protons), 1.64 (p, *J*=7.3 Hz, 2H, β-$\boldsymbol{H_2}$), 1.23–1.36 (m, 20H, alkyl methylene protons), 0.88 (t, *J*=6.9 Hz, 3H, C$\boldsymbol{H_3}$). Abbreviations: s - singlet, t - triplet, m - multiplet, p - pentet.

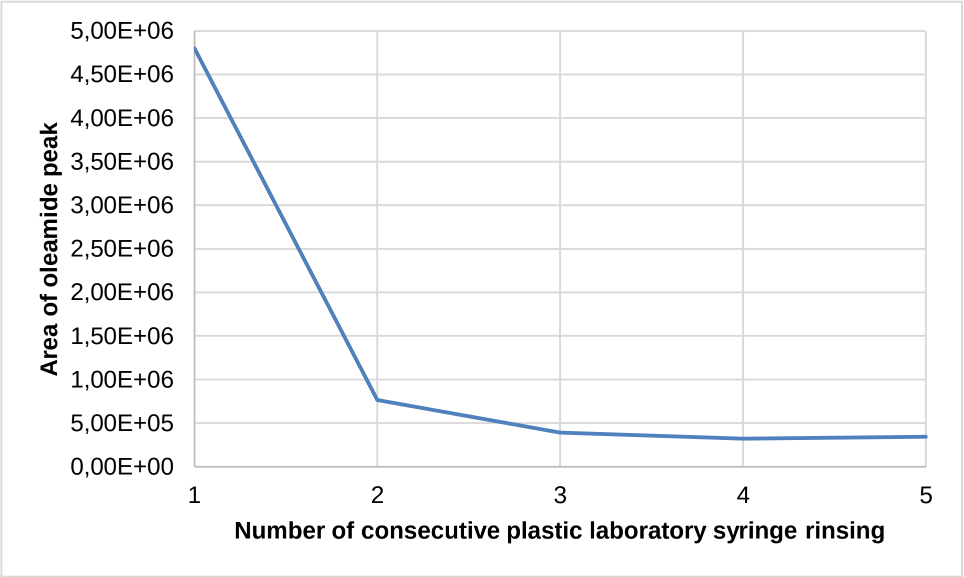

Figure S4. Decrease of a chromatographic peak area of oleamide with the number of consecutive rinsings of a 5 mL plastic syringe with methanol.

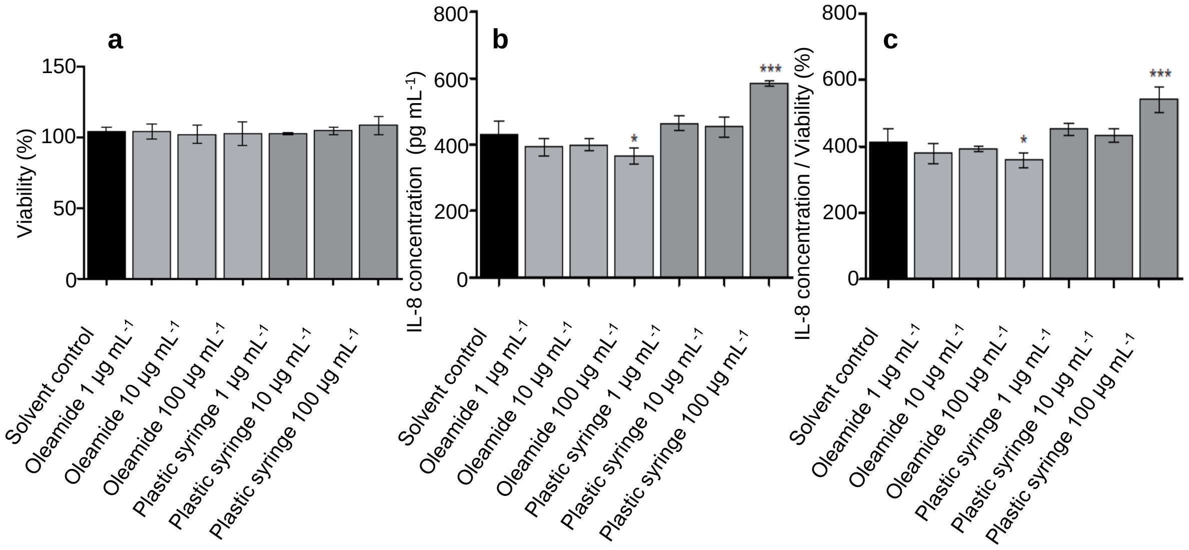

Figure S5. Dose-dependent viability and anti-/pro-inflammatory effects of oleamide and plastic syringe extract in THP-1 monocytes.

THP-1 monocytes were incubated with oleamide, plastic syringe extract or vehicle (70% methanol), followed by stimulation of TLR4 activity with LPS-EB. **a**: Viability (Alamar Blue assay) is displayed in % of viability of untreated cells. **b**: IL-8 concentration in pg mL^{-1} (ELISA). **c**: IL-8 secretion (**b**) divided by viability (**a**). All figures: Data represent means ± SD (*n*=3); unpaired t-test with *p<0.05, ***p<0.0001 (**b**); *p<0.05, ***p<0.001 (**c**) compared to respective methanol control.

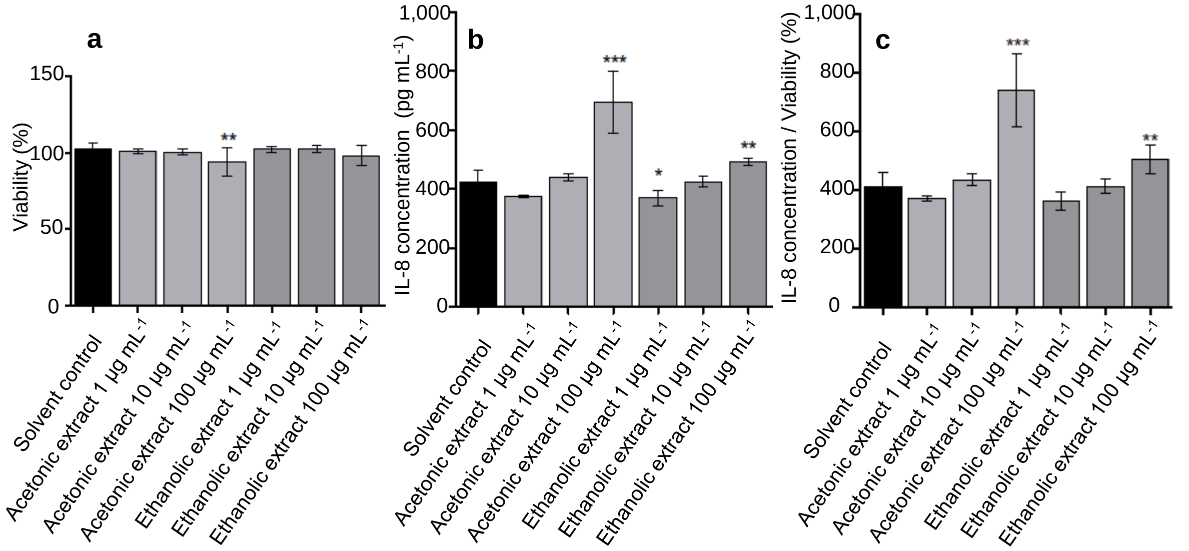

Figure S6. Dose-dependent viability and anti-/pro-inflammatory effects of Japanese knotweed rhizome ethanolic (70%$_{(aq)}$) and acetonic (70%$_{(aq)}$) extract in THP-1 monocytes.

THP-1 monocytes were incubated with Japanese knotweed rhizome ethanolic (70%$_{(aq)}$) and acetonic (70%$_{(aq)}$) extract or vehicle, followed by stimulation of TLR4 activity with LPS-EB. **a**: Viability (Alamar Blue assay) is displayed in % of viability of untreated cells. **b**: IL-8 concentration in pg mL^{-1} (ELISA). **c**: IL-8 secretion (**b**) divided by viability (**a**). All figures: Data represent means ± SD (*n*=3); unpaired t-test with **p<0.01 (**a**); *p<0.05, **p<0.01, ***p<0.0001 (**b**); *p<0.01, ***p<0.0001 (**c**) compared to respective solvent control. Since solvent controls showed similar results, they were summarised to one bar.

Table S1. Tentative identification of other frequently occurring contaminants in the LC-MS chromatograms of labware extracts.

***m/z* (ESI+)**	**t_R (min)**	**Material**	**Tentative identification**	**Ref.**
**228**	8.44	Plastic syringe (5 mL)	Myristamide;<br>Unknown (present in oleamide standard);<br>*N*-butyl-*p*-toluenesulphonamide	1, 2, 3
**242**	11.03, 12.16	Plastic syringe (5 mL)	Tetrabutylammonium ion (TBA)	4
**245**	8.45	Plastic syringe (5 mL)	2,2'-Dihydroxy-4-methoxybenzophenon;<br>Oxotris(propan-2-olato)vanadium;<br>*O*-dianisidine;<br>Azobis(cyclohexanecarbonitrile);<br>Bisphenol A-d16 (BPA-d16)	2
**252**	2.65	Plastic syringe (20 mL)	Benzenesulfonic acid, 4-(diethylamino), sodium salt;<br>Metanilic acid, *N,N*-diethyl-, sodium salt;<br>Pyridinium *p*-toluenesulfonate	2
**254**	10.37	Plastic syringes (5 mL, 20 mL)	Unsaturated amine (present in oleamide standard)	1
**256**	18.41	Plastic syringes (5 mL, 20 mL)	Palmitamide;<br>C14 unsaturated mono alcohol derivative (present in oleamide standard)	1, 3
**268**	14.89	Plastic syringes (5 mL, 20 mL)	Sodium adduct of a fluorinated polymer (PTFE)	5
**273**	18.41	Plastic syringes (5 mL, 20 mL)	Monomethoxytrityl cation (MMT);<br>Polypropylene glycol (PPG) $[M+Na]^+$;<br>Triton $[M+Na]^+$	4, 6
**278**	8.97	Plastic syringe (5 mL)	2-Cyano-3,3-diphenylacrylic acid, ethyl ester;<br>2-Ethylhexyl-4-(dimethyl amino) benzoate	2
**280**	2.65–5.11, 6.31	Plastic syringe (20 mL)	Unknown (present in oleamide standard) – oxidation product?	1, Table 2
**280**	13.49, 14.89	Plastic syringe (5 mL)	Unknown (present in oleamide standard) – oxidation products?	1, Table 2
**282**	23.77	Plastic syringes (5 mL, 20 mL)	*Trans*-oleamide (elaidamide)	7
**284**	6.05, 21.07	Plastic syringe (5 mL)	Stearamide;<br>Oleamide contaminant;<br>Nylon 66	1, 3, 4, 5
**284**	6.05	PVDF filter, Gloves	Stearamide;<br>Oleamide contaminant;<br>Nylon 66	1, 3, 4, 5
**296**	2.65–5.11	Plastic syringe (20 mL)	Oxidation product of oleamide;<br>Octadecyl isocyanate	2, Table 2
**298**	3.10, 6.31	Plastic syringe (20 mL)	Methyl-1-aza-2,2,6,6-tetramethyl-3-cyclohexene ethyl succinate	1

**301**	6.05,<br>21.07	Plastic syringe (5 mL),<br>PVDF filter	[M+Na]$^+$ dibutylphthalate;<br>Oleyol chloride;<br>(Nonylphenyl)phosphate;<br>Sodium (C_{10}-C_{18}) alkyl sulfonate	1, 4, 6, 8
**310**	11.03	Plastic syringe (5 mL),<br>PVDF filter	?	
**314**	5.54	Gloves	*N,N*-Bis-(2-hydroxyethyl)alkyl-(C_{13}-C_{15}) amine C_{15} - 2H;<br>Ethyl stearate	1
**316**	2.65–3.10	Plastic syringe (20 mL)	*N,N*-Bis-(2-hydroxyethyl)-alkyl (C_{15}) amine	1
**328**	1.00–4.5,<br>15.09	Plastic syringe (20 mL),<br>Gloves	Oxidation product of oleamide;<br>(2-Hydroxyethyl)ammonium dodecyl sulphate	2, Table 2
**341**	18.54	H-PTFE	Glycerol monostearate – H_2O;<br>Unknown	1
**345**	15.09	Gloves	Sodium (C_{16}) alkyl sulfonate ($C_{16}H_{33}O_4SNa$)	1
**346**	11.71	Gloves	2,2,4-Trimethyl-1,2-dihydroquinoline (TMDQ dimer)	9
**359**	18.54	H-PTFE	Sodium (C_{17}) alkyl sulfonate	1
**360**	4.35	Gloves	*N,N*-bis-(2-hydroxyethyl)alkyl (C_{13}-C_{15}) amine C_{15} (-2H) + CH_2CH_2OH;<br>$C_{21}H_{45}NO_3$($C_{15}H_{30}N$ $(CH_2CH_2OH)_2$ + CH_2CH_2OH);<br>Erucamide [M+Na]$^+$	1, 4, 6
**361**	25.20	Gloves	Unknown (present in dioctyltin bis(2-ethylhexyl thioglycolate) standard);<br>Triton [M+Na]$^+$;<br>Cyanoacrylate R=C_8H_{17} (UV absorber) AOX-24	1, 4, 6, 10
**368**	8.97,<br>10.43	Plastic syringe (5 mL)	Behentrimonium cation (BTAC-228)	4, 6
**376**	18.54	H-PTFE	Acid Yellow 36;<br>1,3,5-Tris (2,2-dimethylpropanamido)-benzene	2
**378**	25.20	Gloves	?	
**385**	6.31	Gloves	Bisphenol F bis(2-chloro-1-propanol) ether (BFDGE.2HCl)	2
**391**	11.17	Pipette tips	Diisooctylphthalate (DIOP);<br>Di-(2-ethylhexyl) phthalate (DEHP);<br>Di-(*n*-octyl) phthalate (DNOP)	1, 4, 6, 8
**394**	14.10	Gloves	4,4'-Dioctyldiphenylamine	11
**397**	18.54	H-PTFE	Triton $[M_2+H]^+$	4, 6
**403**	2.89, 5.54	Gloves	ATBC impurity;<br>Acetyltributyl citrate;<br>Glycerol monooleate + CH_2O_2;	1

			Unknown	
**415**	2.65	Plastic syringe (20 mL)	Polyethylene glycol (PEG)	4, 6
**455**	8.45	Plastic syringe (5 mL)	Triton, reduced $[M+Na]^+$	4, 6
**476**	10.95	PVDF filter	?	
**511**	17.80	Plastic syringes (5 mL, 20 mL)	1-Piperidinyloxy, 4,4'-[1,10-dioxo-1,10-(decanediyl)bis(oxy)]bis[2,2,6,6-tetramethyl]	2
**563**	21.00	Plastic syringes (5 mL, 20 mL)	4,4'-Bis(2-sulphostyryl)biphenyl, disodium salt	2
**458–986 (∆44)**	9.19	Gloves	?	
**816–992 (∆44)**	26.91	Pipette tips	?	

References:

1. Bradley, E.; Coulier, L. An investigation into the reaction and breakdown products from starting substances used to produce food contact plastics, Report FD 07/01 (2007).
2. Bengström, L. Chemical identification of contaminants in paper and board food contact materials. Dissertation, Technical University of Denmark (2014).
3. Divito, E. B.; Davic, A. P.; Johnson, M. E.; Cascio, M. Electrospray ionization and collision induced dissociation mass spectrometry of primary fatty acid amides. *Anal. Chem.* **84,** 2388–2394 (2012).
4. ESI Common Background Ions, UWPR. Advancing Proteomics http://www.proteomicsresource.washington.edu/protocols05/esi_background_ions.php (2019).
5. Common LC/MS Contaminants, *Agilent Technologies*, September 2003 https://webcache.googleusercontent.com/search?q=cache:ZDs61Ax7yRUJ:https://www.cigs.unimo.it/CigsDownloads/labs/lcmsit/Contaminants%2520Ion%2520Trap.doc+&cd=1&hl=en&ct=clnk&gl=si (2019).
6. Keller, B.O.; Sui, J.; Young, A.B.; Whittal, R.M. Interferences and contaminants encountered in modern mass spectrometry. *Anal. Chim. Acta.* **627,** 71–81 (2008).
7. Bertin, M. J.; Zimba, P. V.; Beauchesne, K. R.; Huncik, K. M.; Moeller, P. D. R. Identification of toxic fatty acid amides isolated from the harmful alga Prymnesium parvum Carter. *Harmful Algae.* **20,** 111–116 (2012).
8. Background Ion List, Rev. A, Waters Connections https://www.waters.com/webassets/cms/support/docs/bkgrnd_ion_mstr_list.pdf (2019).
9. Montaudo, G.; Puglishi, C. Direct pyrolysis of polymers into the ion source of a mass spectrometer (DP-MS) in *Mass Spectrometry of Polymers (*ed. Montaudo, G.; Lattimer, R. P.) 191–246 (Boca Raton, 2002).
10. Bentayeb, K.; Batile, R.; Romero, J.; Nerin, C. UPLC–MS as a powerful technique for screening the nonvolatile contaminants in recycled PET. *Anal. Bioanal. Chem.* **388,** 1031–1038 (2007).
11. Jenke, D. R. Extractables and leachables considerations for prefilled syringes. *Expert Opin. Drug Delivery*. **11,** 1591–1600 (2014).
